# Bovine IgG Prevents Experimental Infection With RSV and Facilitates Human T Cell Responses to RSV

**DOI:** 10.3389/fimmu.2020.01701

**Published:** 2020-08-06

**Authors:** Maaike Nederend, Arthur H. van Stigt, J. H. Marco Jansen, Shamir R. Jacobino, Sylvia Brugman, Cornelis A. M. de Haan, Louis J. Bont, R. J. Joost van Neerven, Jeanette H. W. Leusen

**Affiliations:** ^1^Center for Translational Immunology, University Medical Center Utrecht, Utrecht, Netherlands; ^2^Animal Sciences Group, Department of Cell Biology and Immunology, Wageningen University and Research, Wageningen, Netherlands; ^3^Virology Division, Department of Biomolecular Health Sciences, Faculty of Veterinary Medicine, Utrecht University, Utrecht, Netherlands; ^4^Department of Paediatric Immunology and Infectious Diseases, Wilhelmina Children's Hospital, University Medical Center Utrecht, Utrecht, Netherlands; ^5^FrieslandCampina, Amersfoort, Netherlands; ^6^Cell Biology and Immunology, Wageningen University, Wageningen, Netherlands

**Keywords:** bovine IgG, RSV, immunoglobulin, prophylaxis, T cell activation

## Abstract

Respiratory syncytial virus (RSV) infections represent a major burden of disease in infants and are the second most prevalent cause of death worldwide. Human milk immunoglobulins provide protection against RSV. However, many infants depend on processed bovine milk-based nutrition, which lacks intact immunoglobulins. We investigated the potential of bovine antibodies to neutralize human RSV and facilitate-cell immune activation. We show cow's milk IgG (bIgG) and Intravenous Immunoglobulin (IVIG) have a similar RSV neutralization capacity, even though bIgG has a lower pre-F to post-F binding ratio compared to human IVIG, with the majority of bIgG binding to pre-F. RSV is better neutralized with human IVIG. Consequently, we enriched RSV specific T cells by culturing human PBMC with a mixture of RSV peptides, and used these T cells to study the effect of bIgG and IVIG on the activation of pre-F-pecific T cells. bIgG facilitated *in vitro* T cell activation in a similar manner as IVIG. Moreover, bIgG was able to mediate T cell activation and internalization of pathogens, which are prerequisites for inducing an adaptive viral response. Using *in vivo* mouse experiments, we showed that bIgG is able to bind the murine activating IgG Fc Receptors (FcγR), but not the inhibiting FcγRII. Intranasal administration of the monoclonal antibody palivizumab, but also of bIgG and IVIG prevented RSV infection in mice. The concentration of bIgG needed to prevent infection was ~5-fold higher compared to IVIG. In conclusion, the data presented here indicate that functionally active bIgG facilitates adaptive antiviral T cell responses and prevents RSV infection *in vitro* and *in vivo*.

## Introduction

Respiratory syncytial virus (RSV) infections are a major disease burden in infants and RSV is the second most prevalent cause of death in children, mostly affecting children in low- and middle-income countries (1, 2). It is estimated that 118.200 children died in 2015 because of RSV (1). RSV also is a major seasonal burden to healthcare systems as yearly 3.2 million hospitals admissions are attributed to RSV (1). Efficient protection from RSV will substantially lower healthcare costs as RSV infections are associated with recurrent wheeze during the first years of life in both healthy preterm and term born children (3, 4). Children are especially vulnerable to RSV during the first 6 months of life, when children are mainly dependent on maternal transferred immunity (5). Specifically infants are unable to produce autologous antibodies and maternal antibody titers decrease quickly within the first months (6, 7). It has been shown that breastfeeding reduces the severity and incidence of RSV infections in children (5). Four months exclusive breastfeeding reduces the risk on respiratory and gastro-intestinal tract infections (8, 9). Yet, most children in developed countries fully rely on bovine milk based infant formulas that do not seem to offer a similar level of protection against these pathogens. The current treatment palivizumab is the only available prophylaxis to protect against RSV (10). Palivizumab binds to the post fusion form of the F protein (11). The F protein undergoes conformational changes after RSV binding facilitating fusion with host cells (11).

Human and bovine milk differ in their composition, e.g., bovine milk has lower molecular weight and less diverse milk oligosaccharides than humans. Even though both human and bovine colostrum and milk contain immunoglobulins, bovine milk has a higher concentration of IgG compared to human milk, in which IgA is the most prevalent antibody (12). The most prevalent immunoglobulin isotype in human milk, IgA, is inversely correlated with respiratory tract infections (13). It is hypothesized that a higher IgG concentration in bovine and other ruminant milk is needed because there is no transfer of maternal immunoglobulins during pregnancy in ruminants, making milk the only source for protective immunoglobulin transfer (14). Despite those differences, it has been demonstrated that consumption of raw bovine milk protects infants against respiratory tract infections and the development of allergies and allergic asthma (15–17). Moreover, immunoglobulins from bovine milk are able to detect several common respiratory tract pathogens like RSV (18). Since raw cow's milk confers the risk of transmitting pathogens to infants, milk is normally heat treated before consumption. Heat treatment of milk reduces the protective effect of bovine milk (15, 19, 20). The amount of intact milk protein thus seems to be correlated to the protective potential of bovine milk, indicating that bovine milk loses its protective potential due to denaturation of milk proteins (19, 20).

Although there is no evidence of gastro-intestinal uptake of bovine immunoglobulins, bIgG is shown to interact with the neonatal Fc receptor (FcRn) (21). Furthermore, bIgG has been shown to bind human FcγRII and is able to form immuno-complexes that can mediate activation of monocyte-derived dendritic cells (moDCs) (18, 22, 23). This strongly indicates that supplementation of bIgG to infant formulas could be beneficial for infants.

In the present work, we examined the capacity of purified bIgG to bind RSV, its potential to facilitate RSV-specific T cell responses *in vitro*, and evaluated its prophylactic capacities.

## Materials and Methods

### Cells and Viruses

HEp-2 cells (ATCC) were maintained in Iscove's Modified Dulbecco's Medium (IMDM, Gibco) supplemented with 10% fetal calf serum (FCS), 100 U/ml penicillin and 100 ug/ml streptomycin (Life Technologies) at 37°C and 5% CO_2_. RSV-A2 and RSV-A2-RL-Line19F were propagated in HEp-2 cells, purified by polyethylene glycol 6,000 precipitation, and resuspended in PBS supplemented with 10% sucrose and stored in liquid nitrogen, as previously described in Jacobino et al. (24).

### Bovine IgG

Bovine colostrum was collected from 5 cows within 5 days after calving. The colostrum was cooled and the fat was removed by ultracentrifugation (RCF 100,000^*^G). The fat free milk serum was stored at −20°C until further purification. After thawing the lipid fraction was removed by centrifugation (RCF 23,500^*^G), and acidic colostral whey was prepared to remove casein by precipitation with 1 M HCl at pH 4.2. The precipitated casein was removed by centrifugation, adjusted to pH 6.8 with 1 M NaOH, filtered and diluted in 20 mM sodium phosphate, pH 7.0.

bIgG was then isolated from colostral whey by affinity purification using a column consisting of HiTRap Protein G HP (VWR), followed by acid elution with 0.1 M Glycine-HCl, pH 2,7 and dialysis against PBS. Purity was of bovine IgG was checked by SDS-PAGE.

### Mice

All experiments were approved by the Animal Ethical Committee of the UMC Utrecht (25). Experiments were performed in C57BL/6 mice purchased from Janviers Lab, or in FcRγ–/– C57BL/6, maintained in the Animal Facility of the UMC Utrecht, or in mFcγR I/II/III/IV–/– C57BL/6 mice, kindly provided by Dr. S.J. Verbeek (LUMC, The Netherlands). Mice were aged 8–20 weeks at the start of the experiments, and littermates were used as controls.

### Binding to RSV-Infected Cells

HEp-2 cells were cultured to 70–80% confluency in T75 flasks and infected O/N with 1 × 10^8^ PFU RSV-A2 or RSV-A2-RL-Line19F at 37°C and 5% CO_2_. Cells were trypsinized and 1 × 10^5^ cells/well were seeded in 96 well V bottom plates (Greiner bio-one). Serial diluted bIgG, IVIG and palivizumab were allowed to bind for 45 min on ice and detected with αhIgG-RPE or αbIgG-Alexa647 for 45 min on ice. Antibody binding was analyzed by flow cytometry (BD bioscience, Canto II and FACS Diva software). Relative binding was calculated by correcting for the total infection of the different RSV strains detected by anti-RSV glycoprotein (Merck).

### RSV Neutralization Assay

RSV-A2 or RSV-A2-RL-Line19F (MOI 2) was pre-incubated in IMDM supplemented with 1% FCS in the presence or absence of antibodies for 1 h at 37°C. HEp-2 cells (1 × 10e5 cells) were added and incubated for 1 h at 37°C and 5% CO_2_. Cells were washed and incubated 24 h in fresh medium at 37°C and 5% CO_2_. Cells were trypsinized and infection was stained with 1 ug/ml palivizumab (MedImmune) and 200 times diluted αhIgG-Alexa647 (Southern Biotech). Infection was determined with flow cytometry (BD Bioscience, Canto II and FACS Diva software). The percentage neutralization was calculated by setting the MFI of the uninfected and the infected cells at 0% and 100% neutralization.

### Pre- and Post-fusion Protein Binding

96 well maxisorp plates (Nunc) were coated O/N with 100 ng/ml stabilized pre- and post-fusion (F) protein (26–28). In between steps, plates were washed with 0.05% Tween20 in PBS. Plates were blocked with 0.5% gelatin in PBS for 1 h at room temperature (RT). palivizumab, Intravenous Immunoglobulin (IVIG, Nanogam, Sanquin) and bIgG were diluted in PBS and incubated for 2 h at RT. Horseradish peroxidase labeled goat-αhIgG (Jackson) or sheep-αbIgG (Abd Serotec) was used as detection antibody. Plates were developed with ABTS substrate (Roche) and the absorbance was measured at 405 nm with a Multiscan RC (Thermolab Systems).

### Human T Cell Activation

PBMC were isolated from blood of healthy donors by ficoll separation and cultured in RPMI1640 supplemented with 5% human AB serum, 100 U/ml penicillin and 100 ug/ml streptomycin (Life Technologies) for 14 days at 37°C and 5% CO_2_, 100 ng/ml PepMix RSV (JPT) was added to enrich for the RSV specific T cells. 10 U/ml Interleukin-2 (IL-2) was added to the culture after 7 days. Autologous monocytes were isolated from the PBMC fraction using CD14 magnetic beads (Miltenyi Bioscience) and used as antigen presenting cells. RSV-specific enriched T cells were cell trace violet labeled and incubated with autologous monocytes, pre-F protein and antibodies in Xvivo 15 medium for 5 days at 37°C and 5% CO_2_. T cell activation was determined by the number of CD4+ and CD8+ T cells (αhCD3-PerCP / αhCD4-RPE / αhCD8-PE/Cy7) per 10000 sulfate latex beads (Invitrogen) measured with flow cytometry (BD Bioscience, Canto II and FACS Diva software).

### Binding of bIgG to Murine FcyReceptors

Bone marrow derived macrophages and dendritic cells were cultured from wild-type (WT), FcRγ–/–, mFcγR I/II/III/IV–/– C57BL/6 mice as described previously (29). 96-well MaxiSorp plates (Nunc) were coated O/N with 10 ug/ml antibody diluted 0.1M NaHPO4, pH 9. Plates were blocked with 1 % gelatin in RPMI1640 (Gibco) for 1 h at room temperature (RT). Cells were labeled with 20 uM calcein AM (Invitrogen) for 30 min at 37°C. 1.5 × 10e5 labeled cells/well were allowed to bind to the coated wells for 45 min at 37°C in 0.1% gelatin in RPMI1640. Binding was defined after several washes with 0.1% gelatin in RPMI1640 and measured (excitation 485 nm, emission 527 nm, ThermoFischer Scientif Fluoroskan Ascent FL) calculated compared to the initial fluorescence.

### Internalization Assay Mouse Macrophages

1.5 × 10e8 FITC-labeled *S. aureus* were opsonized with 500 ug/ml IVIG or bIgG or without antibody in 100 μl 1% bovine serum albumin (BSA)-RPMI1640 for 15 min on ice. Washed bacteria were incubated in an effector: target ratio of 1:100 with 1 × 10e5 bone marrow derived WT mouse macrophages in V bottom 96 well plates (Greiner) for 30 min on ice. Cells were washed with 100 ul ice-cold 1% BSA medium and equally divided over 2 wells prior to addition of opsonized bacteria. One part was incubated at 37°C for internalization, while the other part was stained directly. Extracellular immune complexes (IC) were stained with 200x diluted Alexa647 conjugated αhIgG (Jackson) or αbIgG (Jackson) on ice. A decrease in extracellular signal is considered as internalized IC. In addition, cells were washed, fixated with 1% PFA and analyzed by flow cytometry (BD Bioscience, Canto II and FACS Diva software).

### RSV Prophylactic Mouse Model

Female FcRγ–/– C57BL/6 mice or wild-type female littermates of the same age were used. Mice were anesthetized (3–4% isoflurane) and administered intranasal with 50 μl antibody diluted in PBS with a varying dosing (0.2–5 mg/kg) of bIgG or IVIG or with a fixed dose of 5 mg/kg bIgG or 1 mg/kg for a similar prophylactic effect on the viral load. Palivizumab was used at 0.05 mg/kg. Mice were intranasally infected with 3 × 10e6 PFU RSV-A2-RL-Line19F in 50 μl PBS after 24 h. Mice were euthanized by intraperitoneal injection of sodium pentobarbital 5 days post infection. A bronchioalveolar lavage was performed, after inflating the lungs, with 1 ml PBS and used to determine the viral load, as described previously (24).

### Statistical Analysis

Statistical analysis was performed using GraphPad Prism 6 software. An unpaired Student's *t-*test was used to compare mean values between two groups. Statistical analysis for other multiple comparisons was performed using one-way ANOVA. Statistical significance is indicated as follows: ^*^p < 0.05, ^**^p < 0.01, ^***^p < 0.001, ^****^p < 0.0001. All graphs represent mean ± SD of triplicate measurements, unless indicated otherwise.

## Results

### Bovine IgG Binding and Neutralization of RSV

**Figure 1:**
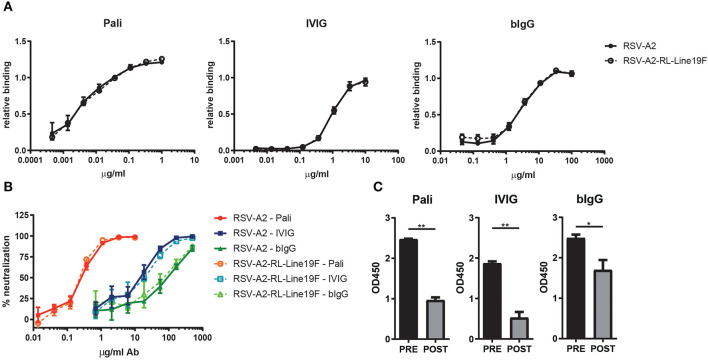
bIgG RSV binding and neutralization. Antibody binding of serial diluted palivizumab (Pali), IVIG or bIgG to RSV-A2 (closed symbols) or RSV-A2-RL-Line19F (open symbols) infected HEp-2 cells. RSV specific binding detected with αhIgG-RPE or αbIgG-Alexa647 and analyzed by flow cytometry. Data corrected for infection rate **(A)**. HEp-2 cells were infected with RSV-A2 or RSV-A2-RL-Line19F which was pre-incubated for 1 h with a serial dilution of palivizumab, IVIG or bIgG. Infection was analyzed by flow cytometry, uninfected cells and no antibody incubation were set as 100 and 0% neutralization, respectively **(B)**. Pre- and post-fusion F glycoprotein specific binding of palivizumab, IVIG and bIgG **(C)**. Median with range of triplicate measurements are shown *P ≤ 0.05; **P ≤ 0.01.

For this study we made use of RSV-A2 and the more pathogenic strain RSV-A2-RL-Line19F, to evaluate whether the binding of purified bovine colostrum IgG (bIgG) and purified human plasma IgG (IVIG) is equal between both strains. HEp-2 cells were infected with RSV and dose-dependent binding of bIgG and IVIG was analyzed. Binding was compared to the clinically used antibody palivizumab (human IgG1 against RSV F protein). bIgG bound to the RSV-A2 infected cells, as shown previously, starting from a concentration of 1.2 μg/ml bIgG. Binding of bIgG was equal to cells infected with both RSV strains, similar to what was observed with IVIG and palivizumab (Figure 1A).

RSV-specific antibodies, like palivizumab, are known to neutralize RSV and are able to prevent infection in children. To evaluate the *in vivo* protective capacity of bIgG we aimed to use the more pathogenic strain RSV-A2-RL-Line19F, however we first wanted to compare the *in vitro* neutralizing capacity of bIgG between RSV-A2-RL-Line19F and the less pathogenic RSV-A2 strain. Previously, we have shown that bIgG is capable of preventing RSV-A2 to infect HEp-2 cells *in vitro* (18). Therefore, both RSV strains were pre-incubated for 1 h with a serial dilution of palivizumab, IVIG or bIgG. Infection of HEp-2 cells was allowed for 1 h at 37°C and cells were washed three times in fresh IMDM medium after incubation to prevent binding of the anti-RSV antibodies to the infected cells and thereby masking the F protein expression of the cells. Infection was analyzed after 24 h by flow cytometry and the neutralization capacity of the antibodies were calculated. All antibodies were capable of neutralizing RSV and preventing infection, as shown previously. The neutralization capacity of all antibodies was equal between both RSV strains (Figure 1B).

### Binding of Bovine IgG to Pre- and Post-fusion F Protein

The RSV fusion glycoprotein (F-protein) is a class I viral fusion protein that is involved in the fusion of the virus the host cell. It undergoes a conformational change from the pre-fusion state to the post-fusion state during viral entry. Antibodies directed against pre-fusion F show a higher neutralization capacity than antibodies directed against post-fusion F (27, 30). Specific binding to plate-bound stabilized pre- and post-fusion F was determined. bIgG was found to recognize both the pre- and the post-fusion F (Figure 1C). The ratio of pre- vs. post-fusion F specific antibodies was higher for palivizumab and IVIG, but bIgG still recognized the pre-fusion state better than the post-fusion state.

### Facilitation of Human RSV-Specific T Cell Activation by Immune Complexes of RSV With hIgG and bIgG *in vitro*

**Figure 2:**
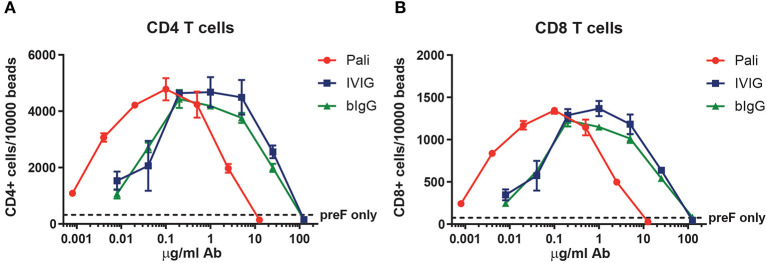
Human T cell activation by RSV prefusion protein-bIgG immunecomplexes. Enriched RSV specific human T cells from healthy donors were incubated with autologous monocytes and immunecomplexes generated by pre-incubating prefusion protein (preF) and a serial dilution of palivizumab, IVIG or bIgG. Activation was determined by the number of CD4 T cells **(A)** or CD8 T cells **(B)** per 10,000 sulfate latex beads with flow cytometry.

Bovine IgG can engage the human FcγRII on myeloid cells when it is bound simultaneously to RSV. These RSV-bIgG immunecomplexes (IC) can be internalized by FcγRII expressing antigen presenting cells (APC) like monocyte-derived dendritic cells (moDC's). To study whether this uptake can result in antigen presentation and thereby leading to activation of the adaptive immune system, a human T cell activation assay was performed. PBMC from healthy donors were enriched for their RSV specific T cells with a RSV peptide mix. Autologous monocytes were used as APC and co-cultured with the RSV specific T cells and IC, formed by co-incubation of pre-fusion F and palivizumab, IVIG or bIgG in titrated concentrations for 5 days. T cell activation was determined by the proliferation of CD4 and CD8 T cells. IC formed with palivizumab showed optimal activation of both CD4 and CD8 T cells with IC formed with 0.1 μg/ml antibody. The curve of bIgG and IVIG looked highly similar, however with an optimum between 0.2 and 1 μg/ml antibody (Figure 2).

### FcγR-Dependent Binding and Internalization of Bovine IgG by Murine Macrophages and Dendritic Cells

**Figure 3:**
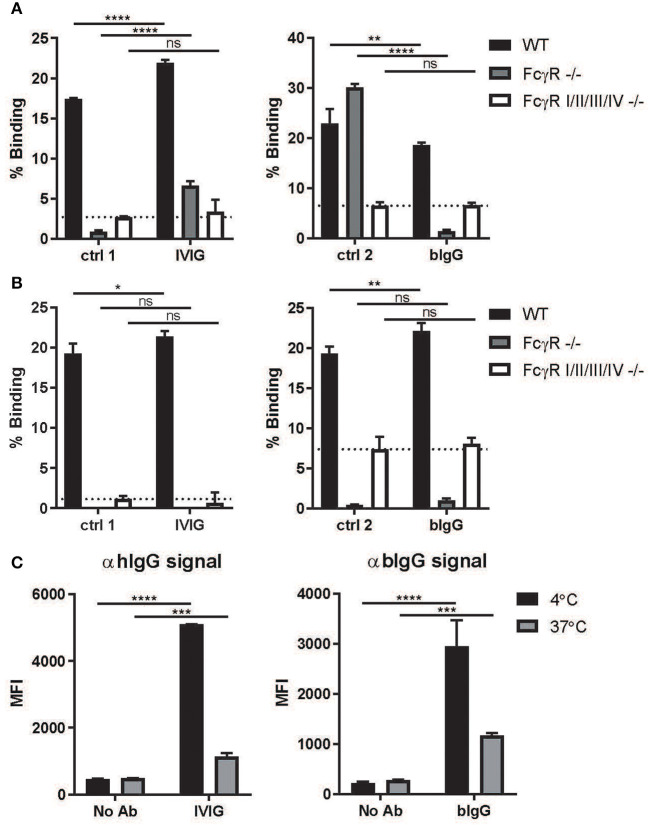
Binding and internalization by activating murine FcγR. Plates were coated with 10 ug/ml IVIG or bIgG and incubated with calcein labeled macrophages **(A)** and dendritic cells **(B)** cultured from bonemarrow of wild-type (WT), FcRγ–/–, mFcγR I/II/III/IV–/– C57BL/6 mice. Binding was compared to human IgG1 (ctrl 1) and mouse IgG1 (ctrl 2) (*N* = 3). FITC labeled S. aureus were opsonized with or without IVIG or bIgG and incubated with WT mouse bonemarrow derived macrophages at 4°C (binding), samples were equally divided and one part was incubated at 37°C for internalization. Extracellular immunecomplexes were determined by Alexa647 conjugated αhIgG or αbIgG and analyzed by flow cytometry. Decrease in signal is considered as internalization **(C)**. Mean with SD of triplicate measurements are shown. *P ≤ 0.05; **P ≤ 0.01; ***P ≤ 0.001; ****P ≤ 0.0001.

To investigate whether bIgG can contribute to the prevention and clearance of RSV *in vivo*, we used a murine RSV challenge model. The *in vitro* data with bIgG and human immune cells suggested that there could be a contribution of active clearance by FcγR-expressing immune cells in the elimination or RSV. bIgG is capable to bind the human activating FcγRIIa, but mice do not express the activating FcγRIIa but only the inhibitory FcγRIIb. Therefore, we first examined whether bIgG could bind murine FcγR. Calcein labeled macrophages and dendritic cells, cultured from bone marrow of wild-type (WT) mice, showed binding to plate bound IVIG and bIgG (Figures 3A,B). Using cells from the FcγR I/II/III/IV knock out (KO) mouse, lacking expression of all the FcγR, resulted in no binding to IVIG and bIgG equal to the control antibodies. In contrast, the cells of the FcRγ KO mice were still able to bind the control antibody mIgG1 and partly to IVIG via the inhibitory receptor FcγRII, the only FcγR expressed by these mice (Figures 3A,B). However, bIgG does not bind to cells of the FcRγ KO mice, demonstrating that binding to bIgG to murine macrophages and dendritic cells is FcγR dependent and only occurs with the activating FcγR. Blocking experiments could not reveal whether one or more of the activating FcγR is responsible for this binding (data not shown).

Next, we examined whether the binding of bIgG to murine FcγR can also induce internalization, as prerequisite for efficient clearance and induction of a memory T cell response. FITC labeled S. aureus were opsonized with or without IVIG or bIgG and incubated with WT mouse bone marrow-derived macrophages. Extracellular IC was determined and compared between 4 and 37°C for internalization (Figure 3C). Both IVIG and bIgG showed a decrease in signal on the outside of the cells indicating that the IC were internalized by the macrophages.

### *In vivo* Prophylactic and FcγR Dependent Activity of bIgG

**Figure 4:**
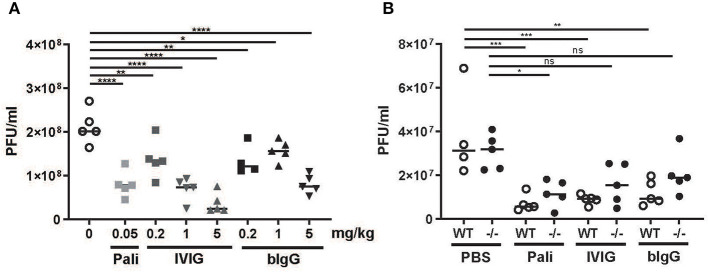
*In vivo* prophylactic activity of bIgG in WT and FcRγ–/– mice. Wild-type (WT) C57BL/6 mice were prophylactically treated with a titration of bIgG or IVIG **(A)** 24 h prior to intranasal infection with 3 × 10e6 PFU RSV-A2-RL-Line19F. RSV load was determined in bronchoalveolar fluid 5 days after infection. The contribution of FcyR was compared in WT (open circles) and FcRγ–/– mice (closed circles) with prophylactic treatment resulting in similar viral load in WT (5 mg/kg bIgG/1 mg/kg IVIG/0.05 mg/kg) **(B)**. *P ≤ 0.05; **P ≤ 0.01; ***P ≤ 0.001; ****P ≤ 0.0001.

The protective capacity of bIgG was further studied in a prophylactic RSV mouse model. A dilution series of bIgG or IVIG and one dosage of palivizumab was administered intranasally 24 h prior to RSV challenge. IVIG was able to reduce viral load in a concentration dependent manner, while bIgG protected against RSV infection in the airways only at the highest dose (Figure 4A). To investigate the underlying mechanism of this protection, we compared the protective effect in WT mice to the effect in FcRγ KO out mice. For optimal comparison between the antibodies, we chose the lowest concentration of antibody that resulted in a protective effect in Figure 4A. The level of infection was equal in the PBS treated mice between the WT and the mice lacking the activating FcγR (Figure 4B). RSV infection was decreased in all treated WT mice with similar levels between the different antibodies. The decrease in viral load was less in the FcRγ KO mice, indicating a role for the activating FcγR, next to the prophylactic neutralizing effect of the antibodies.

## Discussion

In this paper, we have demonstrated that bovine IgG binds to two different strains of human RSV, facilitates the activation of RSV-specific T cells, and reduces viral load with RSV in a prophylactic RSV *in vivo* mouse model.

Bovine IgG is able to recognize pre- as well as post-fusion F protein of RSV, although in a lower pre- to post-fusion F protein binding ratio then IVIG and palivizumab. Despite the fact that binding to pre-fusion F protein is associated with a higher neutralization capacity than antibodies that bind to post-fusion F protein, IVIG and bIgG showed a similar neutralization capacity *in vitro*. It has to be noted that for palivizumab lower concentrations are needed to neutralize RSV compared to both IVIG and bIgG. Since IVIG an bIgG are both polyclonal antibodies, it was expected that higher concentrations would have been needed to reach a similar neutralization compared to the monoclonal antibody palivizumab. Here, neutralization was only tested in RSV-A strains and not in B strains. Since the F protein is highly conserved between RSV A and B strains, it is likely that bIgG is able to bind and neutralize RSV-B strains as well (31). Moreover, we observed that bIgG was also able to neutralize the more pathogenic strain RSV-A2-RL-Line19. Bovine IgG is able to recognize pre- as well as post-fusion F protein of RSV, although in a lower pre- to post-fusion F protein binding ratio then IVIG and palivizumab. Bovine IgG is directed against the bovine RSV. As the prefusion protein of bovine RSV is not identical to the human RSV, as the homology between human and bovine F protein is about 80% (32), it is expected that bIgG has a lower affinity for human RSV pre fusion protein than IVIG and a monoclonal antibody raised against human pre F protein. In addition, cows are often vaccinated against RSV. These vaccines contain attenuated bovine RSV, for example inactivated with formalin (33). It is known that the pre F protein is not stable, and disappears from the RSV surface upon formalin fixation (34). These observations may explain why bovine IgG binds to a lesser extent to human RSV pre F protein.

When RSV-specific T cells are cultured with autologous PBMC's, bIgG and RSV F protein, bIgG as well as IVIG strongly facilitated T cell proliferation, which indicates activation of the adaptive immune system. A similar effect has been described in mice infected with RSV, oral administration of bovine colostrum led to an increased CD8 T cell activity (35). Particularly in RSV infections, the role of T cells is dubious. T cells are, like in other viral infections, required for viral clearance (36). However, it is hypothesized that T cells are also the cause of the vaccination-enhanced disease during the FI-RSV trial (37, 38). Particularly Th2 cells are suspected to play an important role in RSV bronchiolitis immunopathology due to Th2 cytokine release (37). In literature, the activation of T cells by bovine milk has only been evaluated by Xu et al. (35). In this study, activation of CD8+ T cells was observed after oral ingestion of bovine milk (35). However, this increased CD8+ T cell activation in mice was also associated with a lower burden of disease (35). This indicates that the increased T cell activity against RSV that was observed *in vitro*, is likely to only lead to viral clearance without negatively impacting the infection. Moreover, Den Hartog et al. showed that bIgG is capable of recognizing other common respiratory pathogens like influenza and *Haemophilus influenzae* as well, indicating that bIgG might also activate T cell responses to other pathogens (18).

In order to perform prophylactic RSV studies in mice, we first investigated whether bIgG is capable to engage with murine Fc-receptors. We found that bIgG binds murine macrophages and dendritic cells through one or more activating Fc-receptors. We also showed that opsonization by bIgG enabled murine macrophages and dendritic cells to phagocytose *S. aureus*. It has been shown that bIgG is able to form immune complexes that can lead to opsonization of the pathogen. This opsonization is possible mediated by FcRγII as it has been shown that bIgG is able to bind to this receptor (38–400. Moreover, Inhibition of FcRγIIa lead to inhibition of the opsonization of bIgG-HIV-1 immune complexes (39). The *in vivo* prophylactic studies clearly show that both palivizumab, IVIG and bIgG reduced the RSV load in bronchoalveolar fluid. Interestingly, in the FcRγ-/– mice, less protection from RSV was observed for all three antibody groups: palivizumab, IVIG and bIgG. This indicates that also *in vivo* the activating FcγRs are important for RSV antibodies as was described before for palivizumab by Van Mechelen et al. (40). No statistical relevant difference could be found between the mice that either received bIgG or IVIG, indicating that bIgG is not inferior to IVIG in the protection from RSV in mice. A similar protective effect of bIgG was observed in the study performed by Xu et al., demonstrating that oral intake of bovine IgG protected mice from RSV (35).

Conclusively, our data suggest that addition of bIgG may be a novel strategy to increase the protective potential of infant formulas. As stated before, many children are dependent on bovine milk derived infant formulas as they are not breastfed (41). Previous trials evaluating the effect of raw milk or bovine immunoglobulin rich formulas have already shown their efficacy in the treatment of gastro-intestinal infections with rotavirus and *E. coli*. Another trial performed by Loss et al. remarkably showed that children may benefit from raw cow's milk consumption since the raw cow's milk arm showed fewer respiratory tract infections (among which rhinitis and otitis) and fever episodes compared to the processed milk arms (15, 42). However, consumption of raw cow's milk encompasses risk for young children to transmit several pathogens among which tuberculosis, brucellosis and listeria (15). Adding purified bIgG to infant formulas may thus transfer part of the protective effect of raw bovine milk to microbiologically safe infant formulas.

## Data Availability Statement

The raw data supporting the conclusions of this article will be made available by the authors, without undue reservation.

## Ethics Statement

All experiments were approved by the Animal Ethical Committee of the UMC Utrecht.

## Author Contributions

MN designed and conducted experiments and wrote the manuscript. AS wrote the manuscript. JJ and SJ performed experiments. SB, LB, and RN co-supervised the project and critically read the manuscript. CH provided essential materials. JL supervised the project and co-wrote the manuscript.

## Conflict of Interest

RN is an employee of Friesland Campina. The remaining authors declare that the research was conducted in the absence of any commercial or financial relationships that could be construed as a potential conflict of interest.

## Abbreviations

ABTS, 2: 2′-azino-bis(3-ethylbenzothiazoline-6-sulfonic acid)
ADCC: Antibody-dependent cellular cytotoxicity
APC: antigen presenting cells
ATCC: American Type Culture Collection
bIgG: cow's milk IgG
BSA: bovine serum albumin
F protein: RSV fusion protein
FcγR: IgG Fc Receptors
FCS, fetal calf serum FI-RSV: formalin-inactivated respiratory syncytial virus
IC: immune complexes
IMDM: Iscove's Modified Dulbecco's Medium
IVIG: Intravenous Immunoglobulin
KO: knock out
MFI: Mean fluorescence intensity
moDC: monocyte-derived dendritic cells
PBMC: Peripheral blood mononuclear cells
PBS: phosphate buffered saline
PFA: Paraformaldehyde
PFU: Plaque-forming unit
RCF: Relative Centrifugal Force
RPMI: RPMI1640 medium
RSV: Respiratory syncytial virus
SD: standard deviation
WT: wild type.
